# Short-term increase in prevalence of nasopharyngeal carriage of macrolide-resistant *Staphylococcus aureus* following mass drug administration with azithromycin for trachoma control

**DOI:** 10.1186/s12866-017-0982-x

**Published:** 2017-03-28

**Authors:** Ebrima Bojang, James Jafali, Vincent Perreten, John Hart, Emma M. Harding-Esch, Ansumana Sillah, David C. W. Mabey, Martin J. Holland, Robin L. Bailey, Anna Roca, Sarah E. Burr

**Affiliations:** 1 Disease Control and Elimination Theme, Medical Research Council Unit, The Gambia, Fajara, Banjul The Gambia; 2 Institute of Veterinary Bacteriology, Vetsuisse Faculty, University of Bern, CH-3012 Bern, Switzerland; 3 Department of Clinical Research, London School of Hygiene and Tropical Medicine, London, WC1E 7HT UK; 4 National Eye Health Programe, Ministry of Health and Social Welfare, Kanifing, The Gambia; 5 Faculty of Epidemiology and Population Health, London School of Hygiene and Tropical Medicine, London, WC1E 7HT UK

**Keywords:** Trachoma, Azithromycin, Mass drug administration, *Staphylococcus aureus* carriage, Macrolide resistance, iMLS_B_, The Gambia, West Africa

## Abstract

**Background:**

Mass drug administration (MDA) with azithromycin is a corner-stone of trachoma control however it may drive the emergence of antimicrobial resistance. In a cluster-randomized trial (Clinical trial gov NCT00792922), we compared the reduction in the prevalence of active trachoma in communities that received three annual rounds of MDA to that in communities that received a single treatment round. We used the framework of this trial to carry out an opportunistic study to investigate if the increased rounds of treatment resulted in increased prevalence of nasopharyngeal carriage of macrolide-resistant *Staphylococcus aureus*. Three cross-sectional surveys were conducted in two villages receiving three annual rounds of MDA (3 × treatment arm). Surveys were conducted immediately before the third round of MDA (CSS-1) and at one (CSS-2) and six (CSS-3) months after MDA. The final survey also included six villages that had received only one round of MDA 30 months previously (1 × treatment arm).

**Results:**

In the 3 × treatment arm, a short-term increase in prevalence of *S. aureus* carriage was seen following MDA from 24.6% at CSS-1 to 38.6% at CSS-2 (*p* < 0.001). Prevalence fell to 8.8% at CSS-3 (*p* < 0.001). A transient increase was also seen in prevalence of carriage of azithromycin resistant (Azm^R^) strains from 8.9% at CSS-1 to 34.1% (*p* < 0.001) in CSS-2 and down to 7.3% (*p* = 0.417) in CSS-3. A similar trend was observed for prevalence of carriage of macrolide-inducible-clindamycin resistant (iMLS_B_) strains. In CSS-3, prevalence of carriage of resistant strains was higher in the 3 × treatment arm than in the 1 × treatment (Azm^R^ 7.3% vs. 1.6%, *p* = 0.010; iMLS_B_ 5.8% vs. 0.8%, *p* < 0.001). Macrolide resistance was attributed to the presence of *msr* and *erm* genes.

**Conclusions:**

Three annual rounds of MDA with azithromycin were associated with a short-term increase in both the prevalence of nasopharyngeal carriage of *S. aureus* and prevalence of carriage of Azm^R^ and iMLS_B_ *S. aureus*.

**Trial registration:**

This study was ancillary to the Partnership for the Rapid Elimination of Trachoma, ClinicalTrials.gov NCT00792922, registration date November 17, 2008.

## Background

Trachoma, caused by ocular infection with the intracellular bacterium *Chlamydia trachomatis*, is the leading infectious cause of blindness worldwide. Mass drug administration (MDA) with the broad-spectrum antibiotic azithromycin is an important part of the World Health Organization’s integrated strategy for trachoma control [1, 2]. This treatment serves to decrease the reservoir of infection, thereby reducing transmission.

There has been increased interest in MDA with azithromycin following the publication of studies conducted in Ethiopia suggesting treatment is associated with a significant reduction in childhood mortality [3, 4]. Calls to expand azithromycin distribution beyond trachoma-endemic countries [5] and a large-scale clinical trial to evaluate the effect of treatment on mortality that is underway in three African countries [6] bring a greater need to document unintended effects of treatment, including the emergence of antimicrobial resistance, which is a global public health concern.

There is, as yet, no evidence to suggest MDA of azithromycin at the community-level leads to increased azithromycin resistance in ocular *Chlamydia trachomatis* infection [7–9]. However, there are data supporting an association of MDA with the emergence of macrolide-resistant *Streptococcus pneumoniae* isolated from the nasopahrnyx, at least in some settings. While studies carried out in Tanzania, Nepal and The Gambia have shown no evidence of such resistance following a single treatment round [10–12], other studies in Tanzania, Nepal and Australia suggest resistance does emerge after just one or two annual rounds of mass treatment [13–15]. Further studies in Ethiopia have documented increased macrolide resistant pneumococci isolated following four rounds of MDA given at 3 month intervals [16] and following six biannual rounds over a period of 3 years [17]. To date, little work has been carried out to assess the effect of MDA with azithromycin on other bacterial pathogens.

*Staphylococcus aureus* colonization is a risk factor for many conditions ranging from skin and soft tissue infections in children to invasive disease such as neonatal sepsis, bacteraemia and endocarditis [18–22]. In West Africa, it has been shown to be a significant cause of invasive disease in young children [23, 24]. However, the potential effect of azithromycin MDA on prevalence of carriage of *S. aureus* including macrolide resistant strains has not yet been explored. In the present study, we used the framework of a clinical trial, which compared the prevalence of active trachoma in Gambian communities that received three annual rounds of azithromycin MDA to that of communities that received a single MDA round [25, 26] to explore whether three rounds of MDA drove increased nasopharyngeal carriage of azithromycin resistant *S. aureus*.

## Methods

### Study design

**Fig. 1:**
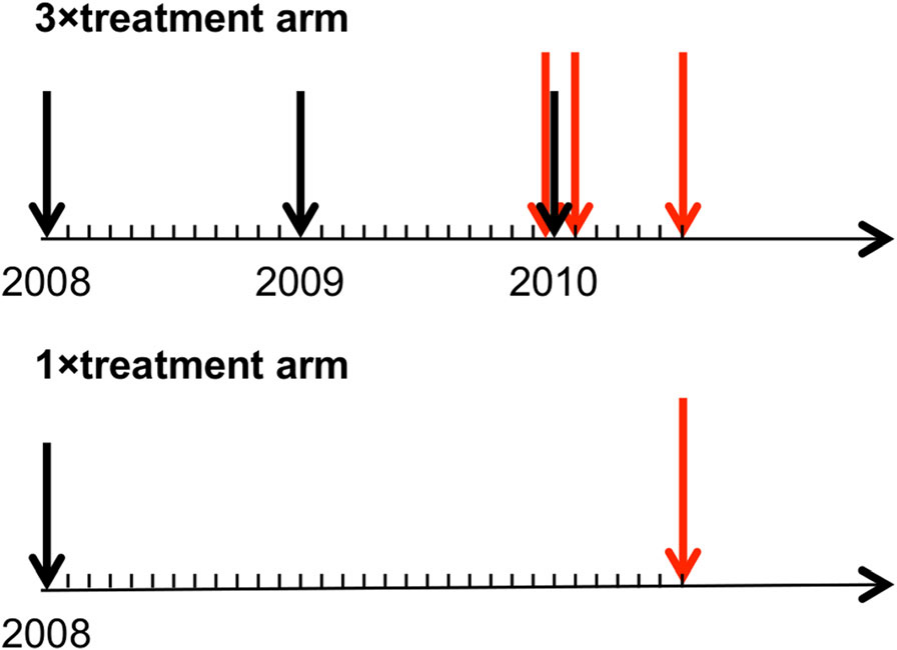
Time-line of treatment and sample collection. MDA with azithromycin is depicted by *black arrows*; NPS sample collection is depicted by *red arrows*

**Fig. 2:**
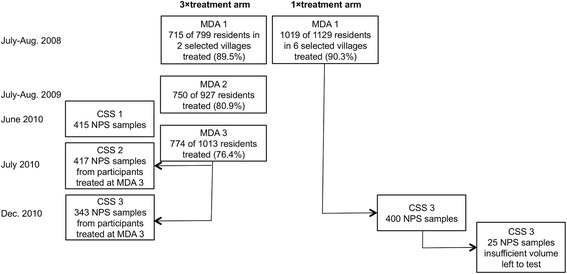
Study profile

The Partnership for the Rapid Elimination of Trachoma (PRET) study (ClinicalTrials.gov NCT00792922) was a cluster randomized controlled trial, the design of which has been described elsewhere [25, 26]. Briefly, the study compared the effectiveness of three versus one round of azithromycin MDA in reducing the prevalence of active trachoma and ocular *C. trachomatis* infection. Treatment was a single oral dose of 20 mg azithromycin per kg to a maximum of 1 g and height was used as a proxy for weight. A pneumococcal carriage study was nested within PRET [12] and was carried out in eight villages that were a part of the larger trial. This included two villages that had been randomized, by the underlying PRET trial, to three annual rounds of MDA (3 × treatment arm) and six villages that received a single treatment round (1 × treatment arm) (Figs. 1 and 2). All villages had also participated in a trial of pneumococcal conjugate vaccine and were part of that study’s control arm, where children under 5 years of age received PCV-7 [27].

Three cross-sectional surveys (CSS) were conducted in the 3 × treatment arm: CSS-1, 1 month prior to the third round of MDA; CSS-2, 1 month following the third round of MDA; and CSS-3, 6 months following the third round (Figs. 1 and 2). CSS-3 also included villages in the 1 × treatment arm that had received one round of MDA 30 months previously (Figs. 1 and 2).

Census data were gathered in the week before CSS-1. All censused children under the age of 15 years and present at the time of sampling were invited to participate. For individuals 15 years of age or older, 150 individuals were randomly selected at each of CSS-1 and CSS-2 and in both treatment arms in CSS-3. Random selection was independent at each CSS.

In the 3 × treatment arm, participation in CSS-2 and 3 was restricted to those who were documented to have received azithromycin during the third round of MDA in July 2010. At each CSS, a nasopharyngeal swab (NPS) was collected from each participant and a questionnaire, pertaining to demographic characteristics, socioeconomic status and recent antibiotic use (within the last 30 days), was conducted. In the present study, we analysed all NPS samples collected as a part of the pneumococcal carriage study [12] to determine the prevalence of *S. aureus* carriage, including carriage of macrolide resistant strains.

### Specimen collection

NPS samples were collected using calcium alginate swabs and inoculated into sterile vials containing skim-milk-tryptone-glucose glycerol transport medium, which has been shown to preserve the viability of respiratory pathogens for up to 12 years when stored at −70 °C [28]. Samples were kept on wet-ice in the field, transferred to a 4 °C refrigerator within 8 h of collection and moved to long-term storage at −80 °C within 24 h of collection. Samples remained in long-term storage at −80 °C with back-up power and twice daily temperature monitoring for 18 months before being cultured for *S. aureus*.

### Laboratory methods

NPS samples were thawed at room temperature and 10 μl transport medium was inoculated onto Mannitol Salt Agar (Oxoid Ltd, Basingstoke, UK) and incubated for 18–24 h at 35 °C. Presumptive *S. aureus* colonies were sub-cultured for purity on a Blood Agar Base No. 2 (Oxoid Ltd, Basingstoke, UK) supplemented with 5% sheep blood (TCS Biosciences Ltd., Botolph Clayton, UK) and incubated under the same conditions. A coagulase agglutination test was performed on well-isolated colonies to confirm the identification of *S. aureus* using the SLIDEX Staph-kit (Biomerieux UK Limited, Basingstoke, UK).

Well-isolated *S. aureus* colonies were suspended in nutrient broth (Oxoid Ltd, Basingstoke, UK) to a 0.5 McFarland standard and plated on Mueller-Hinton Agar (Oxoid Ltd, Basingstoke, UK). Azithromycin (15 μg), erythromycin (15 μg) and clindamycin (2 μg) discs (Oxoid Ltd, Basingstoke, UK) were placed aseptically on each plate with the erythromycin and clindamycin discs spaced 15 mm apart from edge-to-edge. The plates were incubated for 18–24 h at 35 °C.

Sensitivity results were interpreted according to the Clinical and Laboratory Standards Institute guidelines [29]. Azithromycin resistance (Azm^R^) was defined by a zone size ≤13 mm. Macrolide-inducible-clindamycin resistance, also referred to as the inducible Macrolide-Lincosamide-Streptogramin B (iMLS_B_) phenotype, was defined as flattening of the circular zone of inhibition around the clindamycin disc on the side facing the erythromycin disc (D-test) [30]. Constitutive clindamycin resistance (Cli^R^) was defined as a circular zone of inhibition ≤ 14 mm.

Twenty-three *S. aureus* isolates obtained from the 3 × treatment arm at CSS-1 and CSS-2 were randomly selected for analysis of the presence of 117 of the most prevalent and transferable antibiotic resistance determinants found in Gram-positive bacteria using micro-array, as previously described [31].

Laboratory technicians were blind to the treatment arm and the timing of sample collection.

### Data management and statistical analysis

Data were double-entered into an OpenClinica database and the validated data imported into Stata version 12 (StataCorp LP, College Station, Texas, USA) for statistical analyses. Proportions of overall carriage and carriage of antimicrobial resistant *S. aureus* were compared using Chi-Square or Fisher’s exact tests. Logistic regression analyses were further used to identify risk factors for carriage, control for confounders and test for interactions, reporting odds ratios with 95% confidence intervals (CI). CIs and p-values were estimated using clustered robust standard errors to account for within village correlation of participants. Analysis included overall prevalence of nasopharyngeal carriage, prevalence of carriage of Azm^R^ and iMLS_B_ strains and proportions of Azm^R^ and iMLS_B_ strains isolated.

### Ethical review

This study was ancillary to the PRET trial and was approved by The Gambia Government/Medical Research Council Unit, The Gambia Joint Ethics Committee. Written, informed consent was obtained from all participants of the PRET trial and additional written consent was obtained for this ancillary study. In the case of minors, informed consent was obtained from the parent or guardian.

## Results

### Study participants

In the 3 × treatment arm, NPS were collected from 415, 417 and 343 individuals at CSS-1, CSS-2 and CSS-3, respectively. Four hundred participants in the 1 × treatment arm were also sampled at CSS-3. Overall, 25 NPS from the 1 × treatment arm (6.2% of 400 samples) had insufficient volume remaining to conduct the laboratory assays and were excluded from the analysis. Demographic and epidemiological characteristics of the participants, including *S. pneumoniae* carriage [12], are given in Table 1.

**Table 1:** Demographic characteristics of study participants at each cross-sectional survey

	Survey^a^			
	CCS-1	CCS-2	CCS-3	CCS-3
Arm				
1 × treatment	--	--	375 (100.0%)	0 (0.0%)
3 × treatment	414 (100.0%)	417 (100.0%)	0 (0.0%)	343 (100.0%)
Age group				
< 10 y	205 (49.5%)	173 (41.5%)	157 (41.9%)	182 (53.1%)
≥ 10 y	209 (50.5%)	244 (58.5%)	218 (58.1%)	161 (46.9%)
Gender (males)	218 (52.7%)	209 (50.1%)	198 (52.8%)	158 (46.1%)
Ethnicity (Jola)	408 (98.6%)	407 (97.6%)	346 (92.3%)	333 (97.1%)
Occupation				
None	185 (45.2%)	162 (39.0%)	135 (38.1%)	169 (50.0%)
Student	148 (36.2%)	149 (35.9%)	128 (36.2%)	90 (26.6%)
Agriculture	76 (18.6%)	104 (25.1%)	91 (25.7%)	79 (23.4%)
Schooling years				
0	260 (62.8%)	258 (61.9%)	240 (64.0%)	242 (71.8%)
(1–3)	72 (17.4%)	67 (16.1%)	58 (15.5%)	36 (10.7%)
(4–6)	51 (12.3%)	48 (11.5%)	11 (2.9%)	25 (7.4%)
> 6	31 (7.5%)	44 (10.6%)	66 (17.6%)	34 (10.1%)
Able to read (yes)	155 (37.4%)	158 (37.9%)	76 (20.3%)	56 (16.3%)
Able to write (yes)	154 (37.2%)	158 (37.9%)	92 (24.5%)	63 (18.4%)
Recent^b^ health centre visit (yes)	14 (3.4%)	12 (2.9%)	18 (4.8%)	30 (8.8%)
Recent^b^ antibiotic use (yes)	2 (0.5%)	2 (0.5%)	6 (1.6%)	4 (1.2%)
Smoker in the household (yes)	264 (63.8%)	273 (65.5%)	113 (30.2%)	166 (48.7%)
*S. pneumoniae* carriage (yes)	180 (43.5%)	80 (19.2%)	182 (48.5%)	157 (45.8%)

^a^Surveys were conducted immediately before the third round of MDA (CSS-1) and at one (CSS-2) and six (CSS-3) months after MDA

^b^Within the last 30 days

### Prevalence of *S. aureus* carriage

Prevalence of nasopharyngeal *S. aureus* carriage at CSS-1 was 24.6% (102/414 participants) (Table 2). One month following MDA, prevalence of carriage in the same study villages increased to 38.6% (161/417; *p* < 0.001) then fell to 8.8% at CSS-3, 6 months following MDA (30/343; *p* < 0.001) (Table 2). In the 1 × treatment arm, prevalence of *S. aureus* at CSS-3 was similar to the 3 × treatment arm (6.7% versus 8.8%, *p* < 0.295) (Table 2).

**Table 2:** Prevalence of *S. aureus* carriage over time and between treatment arms

Arm	Survey	N	n (%)	Crude OR (95% CI)	p-value	Adjusted OR^a^ (95% CI)	*p*-value
Over time							
3×	CSS-1	414	102 (24.6)	1		1	
3×	CSS-2	417	161 (38.6)	1.92 (1.66–2.23)	<0.001	1.94 (1.68–2.24)	<0.001
3×	CSS-3	343	30 (8.8)	0.29 (0.24–0.36)	<0.001	0.30 (0.25–0.35)	<0.001
Between treatment arms							
1×	CSS-3	375	25 (6.7)	1		1	
3×	CSS-3	343	30 (8.8)	1.34 (0.66–2.74)	0.419	1.47 (0.72–3.00)	0.286

^a^adjusted for age and gender

### Prevalence of antibiotic resistant *S. aureus*

In the 3 × treatment arm, prevalence of carriage of azithromycin resistant (Azm^R^) strains at CSS-1 was 8.9% (37/414) (Table 3). This rose significantly to 34.1% at CSS-2, 1 month post MDA, (142/417; *p* < 0.001) then fell back to previous levels (7.3%, 30/343) at CSS-3, 6 months post MDA (*p* = 0.471, in comparison to CSS-1). There was no evidence of constitutive clindamycin resistance (constitutive Macrolide-Lincosamide-Streptogramin B or cMLS_B_ phenotype) in the 3 × treatment arm at either CSS-1 or CSS-2. A single Cli^R^ isolate was found at CSS-3. Prevalence of carriage of iMLS_B_ *S. aureus* was 5.8% (24/414) at CSS-1 (Table 3), increased to 30.7% (128/417) at CSS-2 (*p* < 0.001) and fell back to previous levels (5.8%, 20/343) at CSS-3 (*p* = 0.673) (Table 3).

**Table 3:** Prevalence of azithromycin-resistant (Azm^R^) and macrolide-inducible clindamycin resistant (iMLS_B_) *S. aureus* isolates over time and between treatment arms

Survey	Total	Resistant (%)	Crude OR (95% CI)	p-value	Adjusted OR^a^ (95% CI)	*p*-value
Overtime						
Azm^R^						
CSS-1	414	37 (8.9)	1		1	
CSS-2	417	142 (34.1)	5.26 (4.95–5.59)	<0.001	5.28 (4.95–5.64)	<0.001
CSS-3	343	25 (7.3)	0.80 (0.45–1.42)	0.447	0.82 (0.47–1.42)	0.471
iMLS_B_						
CSS-1	414	24 (5.8)	1		1	
CSS-2	417	128 (30.7)	7.20 (3.77–13.76)	<0.001	7.24 (3.72–14.1)	<0.001
CSS-3	343	20 (5.8)	1.01 (0.87–1.16)	0.933	1.03 (0.90–1.17)	0.673
Between treatment arms						
Azm^R^						
1×	375	6 (1.6)	1		1	
3×	434	25 (7.3)	4.83 (1.46–16.06)	0.010	5.22 (1.49–18.34)	0.010
iMLS_B_						
1×	375	3 (0.8)	1		1	
3×	434	20 (5.8)	7.68 (1.80–32.84)	0.006	8.37 (1.89–37.14)	0.005

^a^adjusted for age and gender

At CSS-3, prevalence of carriage of Azm^R^ and iMLS_B_ *S. aureus* strains in the 3 × treatment arm was significantly higher than that in the 1 × treatment arm (7.3% versus 1.6% Azm^R^, *p* = 0.010; 5.8% versus 0.8% iMLS_B_, *p* < 0.005) (Table 3).

### Proportion of antibiotic resistant *S. aureus* isolates

When analysed in terms of the proportion of isolates displaying Azm^R^, the results indicate 36.3% (37/102) of *S. aureus* isolates were resistant at CSS-1. This increased to 88.2% (142/161; *p* < 0.001) at CSS-2 and remained high at CSS-3 with 83.3% of isolates (25/30) displaying resistance (*p* = 0.047) (Table 3). The proportion of *S. aureus* isolates displaying the iMLS_B_ phenotype was 23.5% (24/102), 79.5% (128/161) and 66.7% (20/30) at CSS-1, CSS-2 and CSS-3 respectively (Table 4), suggesting a significant increase following treatment (*p* < 0.001).

**Table 4:** Proportion of azithromycin-resistant (Azm^R^) and macrolide-inducible clindamycin resistant (iMLS_B_) *S. aureus* isolates over time and between treatment arms

Survey	Total	Resistant (%)	Crude OR (95% CI)	*p*-value	Adjusted OR^a^ (95% CI)	*p*-value
Overtime						
Azm^R^						
CSS-1	102	37 (36.3)	1		1	
CSS-2	161	142 (88.2)	13.13 (7.67–22.48)	<0.001	13.01 (7.77–21.80)	<0.001
CSS-3	30	25 (83.3)	8.78 (1.11–69.25)	0.039	8.56 (1.03–71.34)	0.047
iMLS_B_						
CSS-1	102	24 (23.5)	1		1	
CSS-2	161	128 (79.5)	12.61 (8.33–19.09)	<0.001	12.52 (7.90–19.85)	<0.001
CSS-3	30	20 (66.7)	6.50 (3.14–13.44)	<0.001	6.39 (3.22–12.66)	<0.001
Between treatment arms						
Azm^R^						
1×	25	6 (24.0)	1		1	
3×	30	25 (83.3)	15.83 (1.97–127.01)	0.009	15.88 (1.99–126.54)	0.009
iMLS_B_						
1×	25	3 (12.0)	1		1	
3×	30	20 (66.7)	14.67 (2.43–88.41)	0.003	18.83 (3.22–110.05)	0.001

^a^adjusted for age and gender

Arm comparison at CSS-3 indicated a significantly higher proportion of Azm^R^ (83.3% versus 24.0%, *p* = 0.009) and iMLS_B_ (66.7% versus 12.0%, *p* < 0.001) *S. aureus* strains in the 3 × treatment arm (Table 4).

### Antibiotic resistance determinants

In a subset of *S. aureus* isolates (*N* = 23), the presence of antibiotic resistance determinants was assayed using a DNA microarray [31]. Results are shown in Table 5. No macrolide resistant determinants were found in six isolates that were sensitive to both azithromycin and clindamycin. Of five isolates displaying resistance to azithromycin but sensitivity to clindamycin, all were positive for the *msr* gene, which conveys resistance to macrolides and streptogramin B. Twelve isolates were azithromycin resistant and had the iMLS_B_ phenotype and all of these carried *erm* genes [11 *erm*(C) and 1 *erm*(T)], confirming their resistance to the MLS_B_ antibiotics.

**Table 5:** Antimicrobial resistance determinants detected in a random sample of *S. aureus* isolates, given by phenotype

Phenotype	Total	Resistance gene(s) detected								*luk-*PV
		*msr*	*erm*(C)	*erm*(T)	*tet*(M)	*tet*(K)	*dfr*(G)	*nor*A	*bla*Z	
Azi^R^, iMLS_B_	12		11	1		1	2	12	10	4
Azi^R^, Cli^S^	5	5					2	5	5	2
Azi^S^, Cli^S^	6				1	1	3	6	6	4

Other antimicrobial resistance determinants identified included the beta-lactamase gene *bla*Z in 21 of isolates screened (91%), the trimethoprim resistance gene *dfr*(G) in 7 isolates (30%) and tetracycline resistance genes in 2 isolates (9%) [1 *tet*(M) and 2 *tet*(K)]. The *norA* gene, which confers resistance to norfloxacin if overexpressed, was detected in all 23 isolates (100%). The Panton-Valentine leukocidin gene, *luk*-PV, was found in 10 (43%) of the isolates screened.

### Risk factors for *S. aureus* carriage following MDA

One month following MDA in the 3 × treatment arm, pneumococcal carriage (OR = 0.58, 95% CI 0.46–0.72, *p* < 0.001), a recent visit to a health centre (OR = 0.48, 95% CI 0.45–0.52, *p* < 0.001), an occupation in agriculture (OR = 0.2, 95% CI 0.1–0.38, *p* < 0.001) and female gender (OR = 0.75, 95% CI 0.57–1.0, *p* = 0.050) were inversely associated with *S. aureus* carriage at CSS-2 according to the adjusted analysis (Table 6).

**Table 6:** Risk factors for *S. aureus* carriage at CSS-2

Characteristic	N	n (%)	Crude OR (95% CI)	p-value	Adjusted OR^a^ (95% CI)	*p*-value
Pneumococcal carriage						
No	337	135 (40.1)	1		1	
Yes	80	26 (32.5)	0.72 (0.67–0.77)	<0.001	0.58 (0.46–0.72)	<0.001
Age group						
< 10y	173	72 (41.6)				
≥ 10y	244	89 (36.5)	0.81 (0.54–1.2)	0.289	1.24 (0.74–2.07)	0.418
Gender						
Male	209	90 (43.1)	1		1	
Female	208	71 (34.1)	0.69 (0.47–0.99)	0.044	0.75 (0.57–1.0)	0.050
Occupation						
None	162	67 (41.4)	1		1	
Student	149	77 (51.7)	1.52 (1.32–1.74)	<0.001	1.12 (0.73–1.71)	0.613
Agriculture	104	17 (16.4)	0.28 (0.10–0.80)	0.017	0.2 (0.1–0.38)	<0.001
Schooling (years)						
0	258	82 (31.8)	1			
1–3	67	36 (53.7)	2.49 (1.62–3.84)	<0.001		
4–6	48	23 (47.9)	1.97 (0.65–6.02)	0.232		
> 6	44	20 (45.5)	1.79 (1.51–2.12)	<0.001		
Ability to read						
No	259	83 (32.1)	1			
Yes	158	78 (49.3)	2.07 (1.20–3.56)	0.009		
Ability to write						
No	259	83 (32.1)	1			
Yes	158	78 (49.4)	2.07 (1.20–3.56)	0.009		
Recent^b^ health visit						
No	405	159 (39.3)	1		1	
Yes	12	2 (16.7)	0.31 (0.22–0.43)	<0.001	0.48 (0.45–0.52)	<0.001
Recent^b^ antibiotic use						
No	415	160 (38.6)	1			
Yes	2	1 (50.0)	1.59 (0.04–59.03)	0.800		
Ethnicity (Jola)						
No	10	3 (30)	1		1	
Yes	407	158 (38.8)	1.48 (1.04–2.10)	0.028	1.5 (1.09–2.08)	0.014
Active smoker						
No	405	159 (39.3)	1			
Yes	12	2 (16.7)	0.31 (0.03–3.14)			
Smoker in household						
No	144	51 (35.4)	1			
Yes	273	110 (40.3)	1.23 (0.63–2.42)	0.548		

^a^Mutually adjusted for pneumococcal carriage, age, gender, occupation and recent health care facility visit

^b^Within the last 30 days

## Discussion

In order to explore the effect of repeated MDA with azithromycin on the prevalence of carriage of macrolide-resistant *S. aureus* and the proportion of resistant strains, we compared communities receiving one or three annual treatment rounds. Our results indicate that MDA was associated with a significant increase in the prevalence of carriage of Azm^R^ and iMLS_B_ *S. aureus* strains immediately following treatment, which returned to lower levels 6 months later. When comparing treatment arms at CSS-3, the prevalence of carriage of resistant *S. aureus* and proportion of resistant strains were higher in those communities that received three rounds of MDA as compared to a single treatment round.

The observation that the prevalence of resistant strains, in the 3 × treatment arm, decreased between CSS-2 and CSS-3 is consistent with research of macrolide resistant *Streptococcus pneumoniae* following azithromycin MDA that has demonstrated resistant phenotypes have decreased fitness when antibiotic pressure is relieved [32, 33]. It may be that, had we collected additional samples at longer time points following treatment, the prevalence in the 3 × treatment arm may have eventually reached that seen in villages that received only a single round of treatment.

When we examine our results as the proportion of *S. aureus* isolates that display macrolide resistance, rather than the prevalence of carriage of resistant strains, resistance remains high 6 months following the last round of MDA (83.3%) suggesting it takes longer for resistance to wane. However, it is difficult to interpret these results as the overall prevalence of *S. aureus* carriage, in both study arms, at CSS-3 was unexpectedly low. While the absolute numbers of resistant isolates were small, so too were the total number of people found to carry *S. aureus* at that time point. Seasonality may explain, at least in part, the difference in carriage between CSS-2 and CSS-3; increased prevalence of *S. aureus* carriage has been reported in a number of populations during the hot, humid summer months [34, 35] and this is consistent with the timing of our surveys (CSS-1 and CSS2 were conducted in the wet season while CSS-3 was conducted in the dry season). However, other external factors may also have played a role. For example, conducting surveys in the wet season while crops are being planted may result in under representation of able-bodied adults amongst those surveyed.

As the proportion of isolates displaying antimicrobial resistance was high, we chose to validate our findings on a subset of isolates using a microarray designed to detect antimicrobial resistance determinants common to Gram–positive bacteria [31]. The results confirmed the presence of determinants encoding resistance to macrolide, lincosamide and streptogramin B antibiotics. While macrolides are not first-line therapy for *S. aureus* infection in Gambia (treatment would usually be cloxacillin or chloramphenicol), their use is indicated in respiratory disease in the case of penicillin allergy and recurrent skin infection, also in the case of penicillin allergy [36]. They would also be considered in the case of suspected atypical pneumonia. The presence of macrolide resistance therefore, while not a cause for immediate concern, is worth monitoring, especially as 91% of the isolates examined by microarray also carried the *bla*Z gene, suggesting concurrent resistance to penicillins in the population.

The majority of the resistance to macrolides was attributed to the presence of either *msr* or *erm*(C) genes however, one strain contained an *erm*(T) gene. To date, *erm*(T) has been primarily reported in *Streptococcus* species [37–39] and has rarely been identified in *S. aureus* isolates [40, 41] suggesting it may have been acquired under selective pressure. The trimethoprim resistance gene *dfr*(G), which was detected in one third of our isolates is reported to be widespread among *S. aureus* isolates in Africa [42]. Almost half of the isolates that were tested by microarray carried the gene encoding Panton-Valentine leukocidin, a pore-forming cytotoxin that has been associated with skin and soft tissue infections and with community-acquired, necrotising haemorrhagic pneumonia [43, 44].

*S. pneumoniae* colonization in the nasopharynx is thought, by many, to be negatively associated with *S. aureus* colonization and interventions to reduce pneumococcal carriage have been associated with an increase in *S. aureus* carriage and disease in some populations [45, 46]. Plausible molecular mechanisms driving competition between the two bacteria are the pneumococcal pilus, which may allow better adherence of *S. pneumoniae* [47] and hydrogen peroxidase production by *S. pneumoniae*, which inhibits *S. aureus* growth [48]. In our study, *S. aureus* carriage was inversely associated with pneumococcal carriage at CSS-2, 1 month following MDA. One possible explanation for this is that the decrease in *S. pneumoniae* carriage immediately following MDA (Table 1) provided *S. aureus* the opportunity to expand its presence in the nasopharyngeal niche.

This was an opportunistic study that utilised the framework of the PRET trial [25, 26] and the pneumococcal carriage study that was nested within PRET [12] to explore associations between carriage of macrolide-resistant *S. aureus* and azithromycin MDA. As such, it has a number of limitations that could have been avoided had this been a prospective study of *S. aureus* carriage. One of these is the lack of baseline data, collected before any MDA. In communities that received just a single round of MDA (1 × treatment arm), 24% of strains isolated were resistant to azithromycin 30 months following treatment (at CSS-3). Data on carriage of macrolide resistant *S. aureus* in The Gambia are scarce, however, samples collected in 2003–2004 from the same region of country as our study (Western Division, now named Brikama Local Government Area) showed 64% erythromycin susceptibility [49, 50]. This suggests macrolide resistance was no higher in our 1 × treatment arm at CSS-3 that it was in the region prior to azithromycin MDA. A survey of school-going children in Cameroon has also reported 75% susceptibility to erythromycin [51] suggesting our results are similar to levels of resistance in other West African countries.

Another limitation was the use of nasopharyngeal swabs, rather than oropharyngeal or nasal swabs, which may have yielded higher numbers of *S. aureus*. However, there is no evidence to suggest the dynamics of macrolide resistance would differ between these respiratory sites. Sampling at additional time points post-MDA would also have helped us determine for how long the prevalence and proportion of resistant strains is maintained within communities following three rounds of treatment.

## Conclusions

Three rounds of MDA for trachoma control were associated an increase in the prevalence of carriage of Azm^R^ and iMLS_B_ *S. aureus* and in the proportion of isolated strains that were resistant to these antibiotics. While the increase in prevalence of carriage of resistant strains was transient, the increase in proportion of resistant strains was maintained for at least 6 months following the final round of MDA*.* Both the prevalence and the proportion of resistant strains was higher in the 3 × treatment arm than in the 1 × treatment arm. These findings highlight the need for continued antimicrobial resistance monitoring in communities receiving azithromycin treatment at the community-level.

## Abbreviations

Azi^R^: Azithromycin resistance
CI: Confidence interval
Cli^R^: Clindamycin resistance
cMLS_B_: Constitutive Macrolide-Lincosamide-Streptogramin B phenotype
CSS: Cross sectional survey
iMLS_B_: Inducible Macrolide-Lincosamide-Streptogramin B phenotype
MDA: Mass drug administration
NPS: Nasopharyngeal swab
PRET: Partnership for the Rapid Elimination of Trachoma
